# Molecular docking analysis of Omt-A protein model from *Aspergillus flavus* with synthetic compounds

**DOI:** 10.6026/97320630019990

**Published:** 2023-10-31

**Authors:** Maneesh Kumar, Ganesh Chandra Sahoo, Waquar Akhter Ansari, Ajmal Ali Mohammad, Abul Farah Mohammad, Joongku Lee

**Affiliations:** 1 Department of Biotechnology Magadh University, Bodh Gaya- 824231, Bihar, India; 2 ICMR-Rajendra Memorial Research Institute of Medical Sciences, Agamkuan, Patna-800007, Bihar, India; 3 ICAR-Indian Institute of Vegetable Research, Varanasi-221005, Uttar Pradesh, India; 4 Department of Botany and Microbiology, College of Science, King Saud University, Riyadh 11451, Saudi Arabia; 5 Department of Zoology, College of Science, King Saud University, Riyadh 11451, Saudi Arabia; 6 Department of environment and forest resources, Chungnam, National University, Daehak-ro, Yuseong-gu, Daejeon, Republic of Korea

**Keywords:** Aflatoxin, docking, product template, O-methyl-transferase

## Abstract

Aflatoxin is a potent mycotoxin of Aspergillus flavus that has been classified as a Group I carcinogen. O-methyltransferase A (Omt-A) is a critical enzyme in the formation of aflatoxin. It catalyzes the methylation of norsalic acid to form the highly toxic intermediate averantin. The ligand-protein interaction of Omt-A was performed with piperlonguminin and blasticidins. The maximum affinity of -10.6 was found for the 5ICC_A piperlonguminine at site1 (X,Y,Z: -15.282, 21.785, 5.672). Compounds such as Blasticidin S, Neoeriocitrin, Blasticidin S - hydrochloric acid, 6,6''-Bigenkwanin, Pipernomaline, and Eriodictyol were found to have binding features to protein residues, as shown by computational interaction at the molecular level.

## Background:

Fungal pathogens are considered the greatest threat to grain production worldwide. They are the main host of the fungus *Aspergillus flavus* and are severely damaged by this pathogen. Disease-causing fungi are widely considered the greatest threat to the global grain industry. *A. flavus* is a pathogenic fungus, meaning that it causes disease in living things such as humans and animals [1, 2]. It is risky because it has the potential to produce aflatoxins, mycotoxins that are highly toxic and carcinogenic. Naturally occurring mycotoxins, called aflatoxins, do more damage to these grains. It is estimated that mycotoxins contaminate about 25% of the world's staple foods each year [3]. Aflatoxins can cause acute hepatitis and possibly liver damage in animals and humans if not treated (8-10). Aflatoxin B1 (AFB1), aflatoxin B2 (AFB2), aflatoxin G1 (AFG1), and aflatoxin G2 (AFG2) are the four most common forms of aflatoxins, with AFB1 being the most potent mycotoxin [4]. Human carcinogens have been identified and classified by the International Agency for Research on Cancer (IARC).

Hepatocellular carcinoma and immune system suppression are just two of the serious health risks associated with the consumption of plants and foods contaminated with aflatoxins, making the inhibition of Omt-A an attractive target for reducing aflatoxin contamination [5]. The goal of molecular docking is to predict the binding affinity and orientation of small molecules (potential inhibitors) with the target enzyme (Omt-A) [6]. This allows scientists to find compounds that might interact with Omt-A and reduce its activity. Molecular dynamics simulations are performed to study the dynamic behaviour of the enzyme-inhibitor combination over time. This provides information about the stability and interactions between the compound and Omt-A and helps to evaluate the efficacy of the potential inhibitors. Structure-based drug design uses the three-dimensional structure of Omt-A to design or optimize compounds that can specifically target and inhibit its activity [7]. Therefore, it is of interest to document the molecular docking analysis of Omt-A protein model from Aspergillus flavus with synthetic compounds.

## Materials and Methods:

In this research, a retrospective observational study was conducted. This research strategy was chosen because there is a wealth of information on the computational behaviour of antifungal agents against the O-methyltransferase-A (Omt-A) proteins of aflatoxin-producing fungi. The information came from a wide range of sources, including scientific articles, online databases, and interviews. The proliferation of Omt-A in aflatoxin-forming fungi has been studied in the context of the effects of natural antifungal agents. The data were then statistically analysed to draw conclusions. The results of the study served to better understand how these compounds affect Omt-A in aflatoxigenic fungi and, consequently, fungal proliferation.

## Sequence retrieval and homology modeling and structure validation:

The primary amino acid sequence of OmtA was retrieved from the NCBI Protein Database with accession number AAS90104.1. The primary sequence of Omt-A consisted of 418 amino acid residues with a predicted molecular weight of 46.82 kDa using the ProtParam server [8]. This protein sequence was used for homology modeling, computational analysis and protein-ligand interaction prediction to find some novel inhibitors against the protein. The Omt-A amino acid residues of *A. flavus* as query sequence were aligned against the Protein Data Bank (PDB) using NCBI-BLASTP to find homologous sequences. The autologous templates were also later detected using the Swiss Model, HHpred, Phyre, and RaptorX web servers. The best structure was modelled was retrieved as the structure of *Thalictrum flavum* (PDBID: 5ICC chain _A) was selected as the best template. The three-dimensional model protein was refined as a homology model based on several sequences alignment using Clustal Omega. The validity of the structure has been demonstrated using genetic and energetic factors. The powerful mechanics of CHARMM, which adheres to the conventional dynamic cascade technique and was used to study the energy and motion of the structured molecule, greatly improved the 5ICC. PROCHECK was used to investigate the quality and stereo-chemical properties of the model [9]. The necessary information about the totality of amino acid residues in the preferred domains, the additional allowed regions, the generously allowed regions, and the disallowed regions was provided by the Ramachandran Plot.

## Molecular docking analysis:

Molecular docking software predicts the three-dimensional structure of protein-molecule interactions. Docking systems are search algorithms with scoring. Before that we predicted the active site of the target protein (5ICC_ChainA) was analyzed using the online server SCFBio-Active Site prediction (http://www.scfbio-iitd.res.in/dock/ActiveSite.jsp) [10]. The search method identifies the correct 3D geometry of ligands in a target protein. The scoring function estimates ligand-protein interactions by predicting binding affinity. Here we performed molecular docking using SeamDock and SwissDock Server to modulate the hypothesized protein-ligand interaction prediction and efficacy. The molecular binding sites module in SeamDock created a 3D grid of the active sites of target proteins and their interaction with ligands. This module showed the pocket of ligand binding site and amino acids for stereo-chemical studies. The effects of ligand on hydrogen bonding, polar contact, and conformation of the target protein were studied. SeamDock makes it easy to get started with small molecule docking. The web server simplifies uploading ligand and protein data, selecting the docking engine, and analysing the results. This simplifies complex docking calculations without installing or configuring a docking engine. Through interactive visualisation, SeamDock's NGL viewer simplifies docking results. SeamDock's interactive interface simplifies small molecule docking for non-specialists [11]. Drug design and development rely heavily on protein-ligand docking simulation. As a result, it is crucial to create web servers for docking simulations. Swiss Dock is a server that can simulate protein-ligand interactions in a straightforward and sophisticated manner. Swiss Dock is an easy-to-use, all-in-one protein-ligand docking program based on EADock DSS [12].

## Result and Discussion:

**Figure 1:**
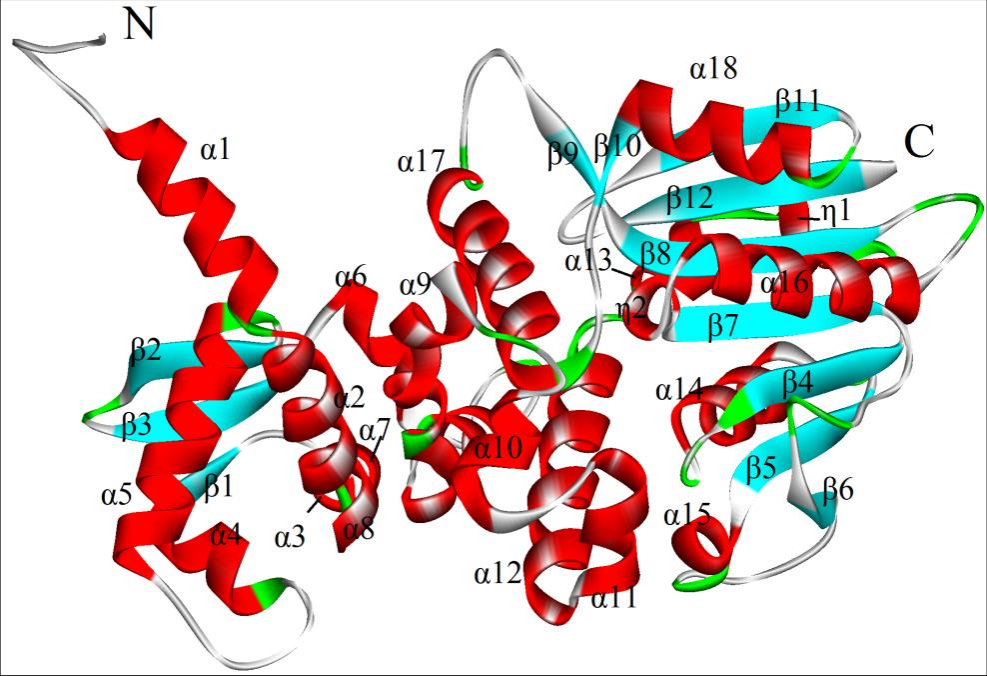
3D Structure of 5ICC_A, modeled protein prepared using Discovery Studio 3.5, α- helix and 310 helices in red and β- sheet in a cyan.

**Figure 2:**
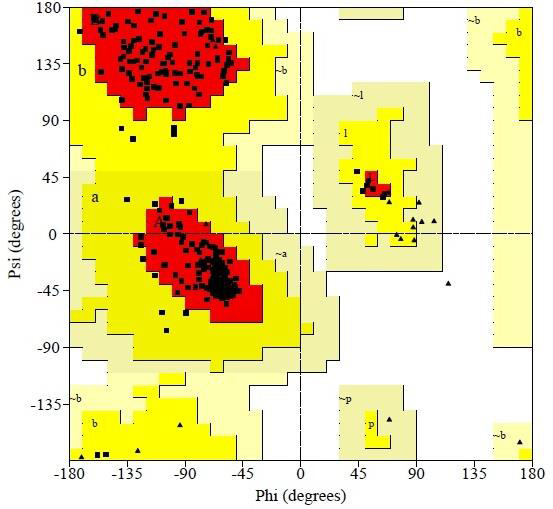
The Ramachandran map for the model protein: red residues are allowed, yellow residues are liberally allowed.

Polyketide-derived aflatoxins are highly carcinogenic in selective aflatoxigenic fungi. Numerous organic intermediates are involved in their biosynthesis, which is mediated by several enzymes. O-methyltransferase (Omt-A) is a crucial enzyme among them, responsible for the formation of O-methylsterigmatocystin [13]. We predicted the tertiary structure because Omt-A lacks a 3D structure, which may be crucial for the generation of anti-OmtA inhibitors [14]. Distant homologues were selected for modeling, validation of different models was performed based on Ramachandran plot using PROCHECK which exhibited 100% residues in most favored regions respectively. Residues in disallowed regions were found 0.0%. The modeled Omt-A structure was improved, and efficient inhibitors were used to target its ligand binding sites (Table 1). The Omt-A model structure was optimized using Discovery Studio version 3.5 (DSv3.5) before molecular docking was performed, and the binding sites were effectively targeted with antifungal ligands. The structure modelled by NMR Chain_A, which is a dimer of 5ICC, was used for the purpose of study. The model's quality is accurately assessed. The monomer chain has five crucial sites, of which sites 1, 2 and 5 are the most suitable for molecular docking. The structure consists of eighteen alpha-helixes (α), two small 310-helixes (η2) and twelve beta-sheets (β) (Figure 1). The amino acid sequence of the protein of interest showed the actual amino acid residues already sequenced. The 5ICC: Chain_A Ramachandran plot exhibited that there is no amino acid residue in the generously allowed regions and in the disallowed regions (Figure 2).

**Figure 3:**
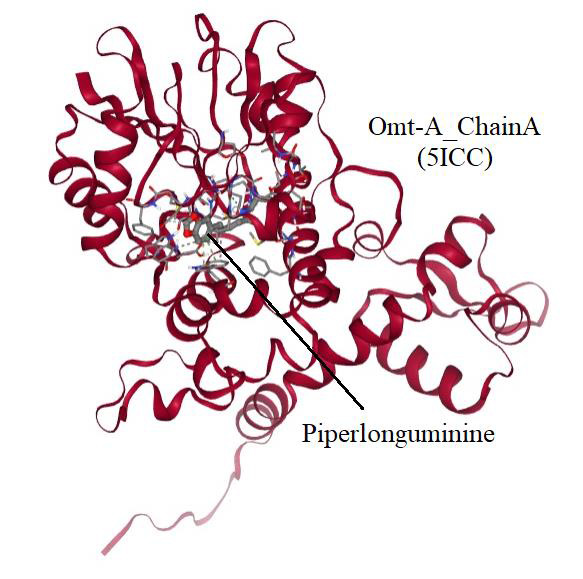
The protein-ligand interaction

We performed a computational assessment of the protein-ligand interaction using SeamDock after certified the binding module (cluster) obtained from SCFBio-Active Site prediction. The interaction the highest affinity of -10.6 (kcal/mol) were obtained for the 5ICC_A piperlonguminine at the site1 (X,Y,Z: -15.282, 21.785, 5.672) interaction (Figure 3).

Gly195 is primary amino acid residue involved as hydrogen bond with the piperlonguminine (CID: 5320621). Trp149, Met166, Leu219 and Trp258 are some crucial amino acid residues behave as hydrophobic interaction with the ligand. Table 1 lists the compounds generated by SeamDock that received the highest affinities including hydrophobic interaction, hydrogen bond and inoinc interction. The compounds with the highest Dock score are neoeriocitrin, blasticidin S hydrochloric acid, 6,6''-benkwanin, pipernomalin, blasticidin S, eriodictyol, and piperlonguminin. In them, neoeriocitrin and blasticidin-S are the most efficient compounds, showing good docking results in many possible positions within the target protein. A more detailed structural view of the relationship can be made using hydrophobic interactions and hydrogen bonding interactions [15]. It is important to note that the blasticidin s that received the highest docking score appeared to block the targeted domain more effectively than any of the others. Further testing, both *in vitro* and *in vivo*, will be performed to evaluate the potential drug-like properties of the selected small molecule.

Piperlonguminine is a compound that holds significant importance in various applications. It is commonly used as a selective agent in molecular biology experiments and cell culture systems. Above all the blasticidin s inhibits protein synthesis by targeting the peptidyl transferase center of the ribosome, making it effective in the selection and maintenance of cells that have been genetically modified to express the blasticidin resistance gene. This compound's significance lies in its ability to enable researchers to selectively grow and study cells that have incorporated the resistance gene, aiding in the understanding of gene function, protein expression, and other related biological processes.

**Figure 4:**
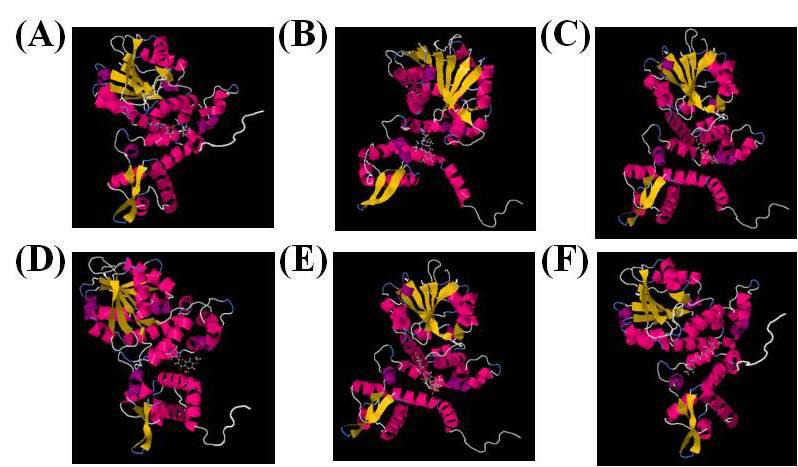
Protein interaction with ligands (A) Blasticidine, (B) Pipernonaline, (C) Genkwanin, (D) Genkwanin, (E) Neoeriocitrin, and (F) Piperlonguminine

The docking result of Swiss Dock is shown in Table 2, where blasticidin actively interacted with the modeled structure with the estimated value of -9.98 kcal/mol. Eriodictyl and pipernonalin also showed interactions with the structure (Figure 4). A number of amino acids at different positions of the Omt-A protein were found to be required for the ligand-protein interface with the compounds of interest. The overlap of blasticidin S with the ligand-binding amino acids of the Omt-A protein is thought to be presented.

## Conclusion:

Piperlonguminine and blasticidin were identified as two of the natural chemicals that actively interact with Omt-A protein. Based on the results of this study of the interaction between the Omt-A protein and synthetic chemicals, it is hypothesized that piperlonguminine may be the most effective compound for controlling aflatoxin.

## Funding:

Authors extend their appreciation to the Researchers Supporting Project Number (RSP2023R306), King Saud University, Riyadh, Saudi Arabia.

## Figures and Tables

**Table 1:** List of Natural Compounds showed their Affinity (kcal/mol) with the amino acids residues took part interaction with respective sites in 5ICC_ChainA.

**Compound name (CID: x) **	**Affinity (kcal/mol)**	**Amino acid residues (Hydrophobic contact)**	**Amino acid residues (Hydrogen bond)**	**Amino acid residues (Ionic interaction)**
Piperlonguminine (CID: 5320621)	-10.6	Trp149, Met166, Leu219,Trp258	Gly195	--
Blasticidin S (CID: 16380)	-8.4	Trp149, Leu219, Trp258	His221, Met239, Cys253, Asp257	Asp150, Asp218
Neoeriocitrin(CID: 114627)	-8.3	Asn147, Phe240	Thr136,Asn 147,Glu262, Gly312	--
6,6'"-Bigenkwanin (CID: 9985404)	-8.1	Thr138, Asn147, Leu219, Pro220, Trp258	Asp150, Thr311	--
Pipernomaline(CID: 9974595)	-7.4	Leu219, Phe240, Trp258	--	--
Blasticidin S- hydrochloric acid (CID:258)	-7.2	Trp149, Lys219	Thr211	Asp257, Asp238
Blasticidins(CID: 439625)	-7.1	Asp289, His 294, Tyr296	Ile287, His337, Ile338, Ser339, Ser343, Ser339, His 337	Asg300, Glu 318, Asp 289
Eriodictyol (CID: 440735)	-7.1	Ala206, Glu231, Ile232	Pro210, Ile212, Gly228, Glu231	--

**Table 2:** Molecular docking with SwissDock server

**Ligands**	**Fitness (kcal/mol)**	**EstimatedΔG (kcal/mol)**
Blasticidine	-2101.83	-9.98
Pipernonaline	-1828.99	-7.8
Eriodictyl	-1821.47	-7.49
Genkwanin	-1798.9	-7.24
Neoeriocitrin	-1685.27	-9.31
Piperlonguminine	-1830.64	-6.87
